# Probabilistic Strategic Conflict-Management for 4D Trajectories in Free-Route Airspace

**DOI:** 10.3390/e22020159

**Published:** 2020-01-30

**Authors:** Javier Alberto Pérez-Castán, Álvaro Rodríguez-Sanz, Luis Pérez Sanz, Rosa M. Arnaldo Valdés, V. Fernando Gómez Comendador, Clemence Greatti, Lidia Serrano-Mira

**Affiliations:** Higher Technical School of Aeronautical and Space Engineering (ETSIAE), Polytechnic University of Madrid, Plaza Cardenal Cisneros, 28040 Madrid, Spain; alvaro.rodriguez.sanz@upm.es (Á.R.-S.); l.perez@upm.es (L.P.S.); rosamaria.arnaldo@upm.es (R.M.A.V.); fernando.gcomendador@upm.es (V.F.G.C.); clemence.greatti@alumnos.upm.es (C.G.); lidia.serrano.mira@alumnos.upm.es (L.S.-M.)

**Keywords:** 4D trajectories, strategic conflict management, conflict detection, conflict probability, temporary-blocking windows, free-route airspace

## Abstract

The expected growth of air traffic in the following decades demands the implementation of new operational concepts to avoid current limitations of the air traffic management system. This paper focuses on the strategic conflict management for four-dimensional trajectories (4DT) in free-route airspace. 4DT has been proposed as the future operational concept to manage air traffic. Thus, aircraft must fulfil temporary restrictions at specific waypoints in the airspace based on time windows. Based on the temporary restrictions, a strategic conflict management method is proposed to calculate the conflict probability of an aircraft pair (that intersects in the air) and to calculate temporary-blocking windows that quantify the time span at which an aircraft cannot depart because one conflict could occur. This methodology was applied in a case-study for an aircraft pair, including the uncertainty associated with 4DT. Moreover, a sensitivity analysis was performed to characterise the impact of wind conditions and speed control on the temporary-blocking windows. The results concluded that it is feasible to propose 4DT strategic de-confliction based on temporary-blocking windows. Although, uncertainty variables such as wind and speed control impact on the conflict probability and the size of the temporary-blocking windows.

## 1. Introduction

The continuous growth of air traffic is expected to double their present values around 2040 [1]. The current system of air traffic management (ATM) presents several inefficiencies and will not be able to manage the air traffic increase. The European macro programme Single European Sky ATM Research (SESAR) addresses the development and implementation of the future ATM system [2]. Among other operational concepts, this paper focuses on the trajectory-based operations (TBO) or four-dimensional trajectories (4DT) and their implementation on a free-route airspace (FRA). 

The 4D trajectory of an aircraft consists of the three spatial dimensions plus time as a fourth dimension, defining each waypoint by position (latitude, longitude and flight level) and time [3]. This operational framework foresees trajectory restrictions regarding time: users will follow trajectories without practically any geographical limitations (according to free-route trajectories), as long as they comply with the planned times. Thus, the main objective of this approach is to ensure an optimal trajectory (free-route trajectory whenever would be possible) by “forcing” the aircraft to accurately meet the time of arrival at designated waypoints in exchange [4]. Most of the proposed methods to model 4DT and solve the aircraft trajectory prediction problem can be categorised into two approaches: deterministic and probabilistic [5]. The traditional approach is based on a deterministic formulation, a mathematical problem that describes the aircraft motion. If some external forces or parameters (e.g., aircraft performance, weather conditions, navigation systems accuracy, traffic regulations) are unknown or cannot be precisely appraised, the probabilistic approach transforms the problem into a stochastic one [6,7].

The TBO concept involves separating aircraft via strategic (long-term) trajectory definition, rather than the currently-practised tactical (short-term) conflict resolution [8]. This aims to increase the air traffic capacity by reducing the controllers’ workload. Nevertheless, real-time measures (over the trajectory) will be required to improve reliability, react to unplanned conditions and thus maintain the expected capacity [9].

Numerous studies focus on conflict detection and resolution in a short-term horizon based on collision avoidance manoeuvres. Several techniques were developed for conflict detection and the projection of the further status of both aircraft; the authors recommend [10,11] for a full understanding of the techniques. Moreover, more complex solutions were developed by different authors based on mathematical techniques [12,13,14]. Particularly, conflict detection in free-flight is not a novel concept [15,16]. Paielli and Erzberger [17] developed the concept of trajectory specification based on pairwise conflict detection and resolution. The authors paved the way with this work for further assessment of 4DT. Barnier and Allignol [18] presented an optimisation problem to estimate the cost of directly solving conflicts in the upper airspace with delays. However, there is few research that focuses on mathematical criteria and probabilistic assessment to appraise the viability of the introduction of 4DT in a FRA.

Typically, conflict detection was tackled based on a static approach. Typically, there exists a cylinder-shaped “forbidden volume or protected airspace zone” around the aircraft defined longitudinally by 5 Nautical Miles (NM) and vertically by 1000 feet (ft.). The conflict area or critical section refers to the whole airspace near an intersection where an aircraft can infringe the separation minima depending on the relative position of other aircraft. Geisinger [19] considered an airspace intersection geometry without any geometrical boundaries. International Civil Aviation Organisation ICAO [20] denoted a conflict area around the intersecting point shaped like a rectangle. Netjasov [21] transformed this conflict area to a critical section as a circle around the intersecting points. Although every geometrical concept is valid, the problem arises when uncertainties are associated with the aircraft movement (stochastic approach). Among different solutions, one of them at the present time is the blocking-area concept [22,23]. The blocking-area allows knowing if an aircraft influences the trajectory of other aircraft and may imply a modification of the other trajectory. When the blocking-area transforms its application from location to time, it evolves to a temporary-blocking window [24,25,26]. In this case, the temporary-blocking window provides the time span an aircraft cannot enter in the critical section because one conflict will arise. Then, it provides temporary restrictions to the operation of an aircraft. The main question arises when it is demanded to know if it is possible to specify temporary restrictions between aircraft departing from different airports based on 4DT for the future scheduling process.

This research provides a probabilistic assessment of the incompatibilities between 4DT depending on the city-pairs. The goal is to provide the temporary-blocking windows on the strategic horizon that limits the departures between different airports. This concept tries to solve the typical approach of solving the conflicts in-flight in order to avoid departure delays [17,27]. However, air traffic is expected to double their values in the following 20 years and, in addition, there is a lack of capacity because of air traffic control (ATC) limitations [28]. The integration of 4DT together with temporary-blocking windows can ensure conflict-free trajectories and, in turn, increase capacity because of a reduction of ATC workload. Therefore, this paper tackles the safe integration of 4DT by strategic conflict management. This approach analyses the impact of uncertainty of 4DT in terms of conflict probability according to temporary restrictions that must be fulfilled by aircraft. A further goal is to calculate the temporary-blocking windows that block the departure of an aircraft depending on another aircraft operating 4D trajectories. 

The remainder of this work is structured as follows. Section 3 introduces the methodology to tackle the problem. Section 3 details the probabilistic assessment for the strategic conflict management and Section 4 describes the process to analyse the conflict probability. Section 5 presents the results in a real case evaluating ideal results with the inclusion of uncertainty parameters. Lastly, Section 6 provides the primary conclusions.

## 2. Methods

### 2.1. Path Modelling for Free-Route Airspace

Currently, airspace users (AUs) define the flight profile based on the predefined routes and waypoints. The operational concept of FRA seeks the path optimisation depending on the AU needs. In this way, the AUs can select the trajectory that best fulfils its operational requirement according to fuel consumptions, aviation charges or flight duration. Herein, it is assumed that full airspace is FRA and, therefore, aircraft will fly according to the shortest route (orthodromic routes) between city-pairs. Equation (1) provides the orthodromic route:

(1) $$sin\left(\lambda -\lambda_{0}\right)=tan\left(\theta_{0}\right)tan\left(\varphi \right)$$

where λ and φ are the longitude and latitude of each point, $\theta_{0}$ is the path course at the equator, and $\lambda_{0}$ the latitude at the equator. In this way, the lateral path is calculated based on the geographical coordinates of the city-pair. Although it is considered the shortest route, the straight route is not the flight with the shortest flying-time in aviation because the shortest flying-time flight is the maximum circle arc. Optimised or loxodromic routes could be as well introduced depending on the AU priorities because the information required for the conflict-detection model is just the 4DT.

According to the vertical profile, the trajectory is defined based on a simple model that entails: a continuous climbing up to Top of Climb (ToC), a cruise level phase (constant speed is assumed) and a continuous descending phase from the Top of Descent (ToD).

Moreover, the 4D trajectory is not characterised by pre-defined waypoints, but it demands new waypoints or “control points” based on distance-separation criterion [3]. Herein, N waypoints are fixed for time-windows requirements of 4DT. They are separated by a constant distance l that is being currently considered from 150 to 200 NM because the path degradation can be assumed [4]. The N waypoints are uniformly distributed from the ToC to the ToD.

The geographical coordinates of the new waypoints are calculated based on Equation (2).

(2) $$sin\left(l\right)=\frac{sin\left(\varphi_{j}-\varphi_{i}\right)}{cos\left(\theta_{i}\right)}$$

where the indicators i and j denote two consecutive waypoints. 

**Figure 1:**
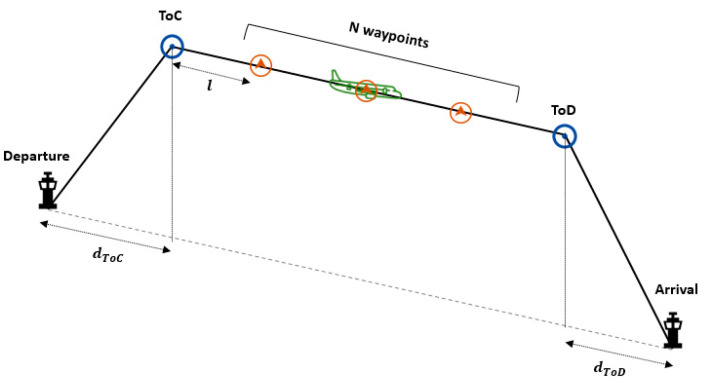
Vertical trajectory profile.

Figure 1 shows that l is the distance between the two consecutive waypoints, t is the time, and $t_{ToC}$, $t_{WP_{i}}$ and $t_{ToD}$ are the time of the aircraft at the ToC, waypoint i and ToD. The generation of trajectories is based on BADA performance model [29].

### 2.2. Conflict-Detection Model

The goal of this module is to develop the conflict-detection model for 4DT by an aircraft pair flying intersecting city-pairs. This model determines the temporary-blocking windows that should apply between departures, according to both trajectories. The temporary-blocking windows calculate the required on-ground time-based separation to ensure separation assurance at the crossing point. This model only considers the case of aircraft departing from different airports. This limitation implies that trailing conflicts are out of the scope of this research. Nonetheless, the authors recommend the work of [21] and [30] that propose different methods to solve this type of conflicts. 

Conflict is the safety metric considered herein because it is one of the most used worldwide. However, to detect the number of conflicts and then the conflict probability means several issues because different features of the conflicts are not considered. Other safety metrics considered by different authors [20,26,31,32,33] are out of the scope of this problem. In future work, to bear in mind other safety metrics could improve the risk assessment of 4DT.

#### 2.2.1. Critical Section

A conflict is defined as the situation where a separation minima infringement will occur. This infringement is modelled herein based on the critical section [34,35]. The critical section ($d_{cri}$) is located around the intersection of two trajectories depending on the separation minima $S_{min}$ and the crossing angle γ.

(3) $$d_{cri}=\frac{S_{min}}{sin\left(\gamma \right)}$$

The best situation is the geometry with a crossing angle γ=90°. As the crossing angle deviates from 90º, both the critical section and the conflict probability increases. Netjasov [21] already investigated the way it can affect and concluded that the crossing angle is a crucial variable to take into account. 

#### 2.2.2. Temporary-Blocking Window

The operational concept of the temporary-blocking window is it calculates on-ground time-based separation on-ground for an aircraft pair ensuring the separation assurance at the crossing point. Based on the position of the aircraft m, there are two critical situations regarding the initial or final points of the temporary-blocking windows. If the aircraft pierces into these two critical situations, a separation minima infringement will occur:

- The initial critical situation occurs when the aircraft m is at the beginning of the critical section, and the n aircraft is leaving.
- The final critical situation occurs when the aircraft m is leaving the critical section, and the aircraft n is entering.

**Figure 2:**
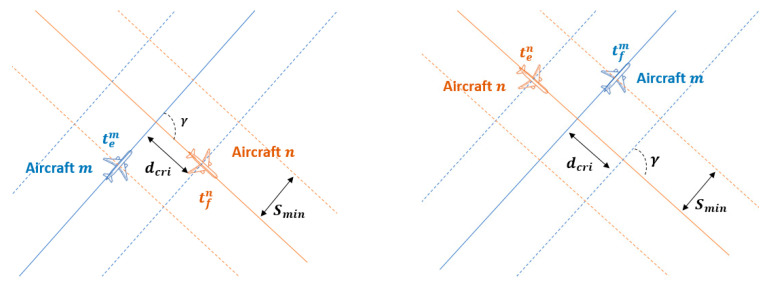
At the left initial critical situation; at the right final critical situation.

Figure 2 represents critical situations.

The initial critical situation is characterised because the aircraft m is located at the instant $t_{e}^{m}$ at the entry point of the critical section. It should be noted that the origin time is the departure time of the aircraft n ($t_{dep}^{n}$). According to the conflict geometry and the operational parameters of the aircraft n:

(4) $$t_{int}^{m}=t_{e}^{n}+\frac{d_{cri}}{v_{n}}$$

where $t_{int}^{n}$ is the expected time for the aircraft n to be at the intersection, and $v_{n}$ is the cruise ground speed. Going backwards in the trajectory of the aircraft m, the initial departing time ($t_{dep_{ini}}^{m}$) can be calculated at which the aircraft m should not depart to not generate a conflict.

(5) $$\begin{array}{c} t_{dep_{ini}}^{m}=t_{e}^{m}-t_{ToC}^{m}-t_{cr}^{m}\\ t_{cr}^{m}=\frac{d\left(t_{e}^{m}\right)-d_{ToC}^{m}}{v_{m}} \end{array}$$

where $t_{ToC}^{m}$ is the expected time of the aircraft m to be located at the ToC, $t_{cr}^{m}$ is the time required from the aircraft m to cover the separation between the ToC and the entry point of the critical section ($d\left(t_{e}^{m}\right)$).

The final critical situation is characterised because the aircraft m leaves the critical section at the time $t_{f}^{m}$ while the aircraft n is piercing into the critical section. According to the geometry of both trajectories, the following condition must be fulfilled:

(6) $$t_{f}^{m}=t_{int}^{n}-\frac{d_{cri}}{v_{n}}$$

The final departing time ($t_{dep_{fin}}^{m}$) at which the aircraft m should not depart in order not to generate conflict is:

(7) $$\begin{array}{c} t_{dep_{fin}}^{m}=t_{f}^{m}-t_{ToC}^{m}-t_{cr}^{m}-\frac{2d_{cri}}{v_{m}}\\ t_{cr}^{m}=\frac{d\left(t_{e}^{m}\right)-d_{ToC}^{m}}{v_{m}} \end{array}$$

In this way, the time span $\left[t_{dep}^{m};t_{dep_{fin}}^{m}\right]$ provides the temporary-blocking windows for the aircraft m between the aircraft m and n. In other words, in the case the aircraft m departs inside the temporary-blocking windows, a conflict can occur between both aircrafts at the critical section. The crossing angle affects the size of temporary-blocking windows by $d_{cri}$. The greater the value of $d_{cri}$, the temporary-blocking windows increases. One issue that should be investigated in further research is the operational application of the temporary-blocking windows. The operational application is crucial because if it considers the application prior to the push-back, the aircraft reduces fuel consumption but new uncertainties are considered. If it considers the location before reaching the departure, it will have a great impact on the queue because of an unfair burden. This research only considers the strategic horizon in which the temporary-blocking windows applies into the allocation of flight plans

## 3. Probabilistic Models

Once the trajectories for both city-pairs are calculated based on FRA, the uncertainties associated with different operational factors are introduced. First, the trajectories are going to be modelled based on the uncertainty associated with 4DT time windows. This is the most complex step because the aircraft must ensure they are going to ensure time windows at specific waypoints based on predefined trajectories. Second, the aircraft evolution suffers uncertainty throughout the stretch between both waypoints, mainly by the wind and speed. The goal of this work is strategic conflict management; therefore, it only has been considered the uncertainty from the last waypoint before the intersection to the next waypoint. In other words, the previous deviations to the last waypoint before the intersection are introduced in the uncertainty at these waypoints.

### 3.1. Uncertainty Associated With 4D Trajectories

The introduction of 4DT implies the fulfilment of an agreement between the AUs and the air navigation service providers (ANSPs). This agreement entails the definition of a set of waypoints throughout the trajectories, in which the pilot must pass through them fulfilling a predefined time window of +/- 2 min the 95% of the time [36]. The main implications of this concept are there is a fair distribution of the responsibility between AUs, ANSPs and the Network Manager (NM) in Europe [37]. Initial validation activities and exercises about 4DT concept confirmed that the trajectory must be adjusted between NM and AUs at the strategic level. Once the 4DT is defined, pilots must ensure that they fulfilled the time-windows requirement supported by ATC. Then, the workload can be increased both pilot and ATC at a tactical level depending on the proper development in the strategic and pre-tactical operational phases.

Herein, the previous waypoints to the intersection are denoted as X for the aircraft m and Y for the aircraft n. 4DT uncertainty at the waypoints can be modelled as μ=0 and σ=60 to ensure 95% (2σ) the time required at a specific waypoint. For instance, the time span the aircraft m must fulfil at waypoint X is $\left[t_{X}^{m}-2\sigma ;\;t_{X}^{m}+2\sigma \right]$. Where $t_{X}^{m}$ is the time of the aircraft m at the waypoint X.

### 3.2. Speed Uncertainty

When both aircraft reach the waypoints X and Y respectively, there is speed uncertainty until the intersection. The planned speed in advance can be different from the operating speed. Once the aircraft crosses a waypoint, it should adjust the speed to fulfil the time windows at the next waypoints. With the aim of seeking different possibilities, two different speed uncertainty modes have been analysed: adjusted and updated speed. These modes are applied independently to both aircraft. 

#### 3.2.1. Mode of Adjusted Speed

This mode adjusts the speed to the average value of the 4DT time windows for the next waypoint. In the case the aircraft reaches the waypoint X beforehand or delayed to the estimated average value ($\bar{t_{X}}$), the speed up to the next waypoint is modified to achieve the next estimated average value. Therefore, in the case the aircraft reaches before the expected time ((Δt<0), it will speed down; otherwise, the aircraft will speed up. The adjusted speed $v^{\prime }$ is calculated as follows:

(8) v′=lΔt+lvΔt=tX¯−tX′

where $t_{X}{}^{\prime }$ is the real-time of the aircraft at the waypoint X. Moreover, the speed modification is constrained up to 5% nominal speed to avoid disproportionate and non-operating speed modifications during the en-route phase.

#### 3.2.2. Mode of Updated Speed

This mode modifies the planned speed with an updating factor α. This updating factor relates the expected average time at the waypoint with the real crossing time, i.e., it updates the speed ($v^{\prime \prime }$) to the real speed the aircraft has operated, and it is expected to continue flying.

(9) $$\begin{array}{c} v^{\prime \prime }=\alpha v\\ \alpha =\frac{\left(\bar{t_{X}}-t_{ToC}\right)}{\left(t_{X}{}^{\prime }-t_{ToC}{}^{\prime }\right)} \end{array}$$

One operational problem of this concept is that speed adjustments increase operational costs for airlines. However, 4DT concept may involve this type of requirements to ensure the achievement of time windows.

### 3.3. Wind Uncertainty

The wind is the most important source of uncertainty in the planning of trajectory because of the inaccuracy of the forecasts. The wind is modelled following two assumptions: (1) The wind remains constant throughout the trajectory, and (2) the wind varies at different geographical areas. However, one of the assumptions of this study is that we only consider the effect of the wind in the ground speed (GS) and not in the path adhesion. The aircraft deviation with regard to the lateral path due to the wind is not considered. Then, the real speed is calculated as:

(10) GS=v−Wcos(β)

where W is the wind and β is the angle of the wind concerning the aircraft heading. Convective weather can generate hardest delays or non-compliance of time windows because aircraft would have to avoid these areas. However, this type of non-standard events are not considered and requires further research.

## 4. Probabilistic Assessment

**Figure 3:**
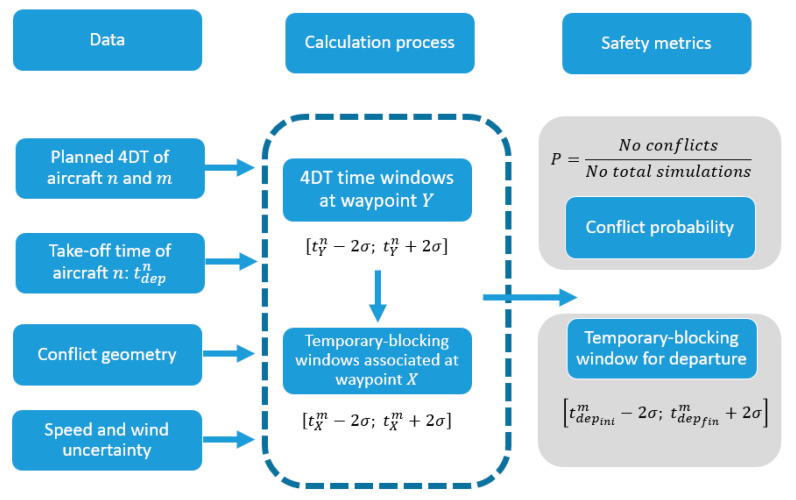
Process of the strategic conflict management based on 4D trajectories.

The final result of this work is two-fold: to determine the conflict probability in 4DT and to estimate the temporary-blocking windows at which an aircraft should not depart. Figure 3 shows the process to calculate the temporary-blocking windows that allow strategic conflict management with 4D trajectories. 

The process to detect conflicts and to determine the temporary-blocking windows for the aircraft m is as follows:

1. It is known the departing time of the aircraft n ($t_{dep}^{n}$) and the planned 4DT of both aircraft.
2. It is calculated the 4DT time windows of the aircraft n at the closest waypoint (Y) to the intersection $\left[t_{Y}^{n}-2\sigma ;\;t_{Y}^{n}+2\sigma \right]$.
3. According to the equations described in Section 2.2, the temporary-blocking window associated with aircraft m at the waypoint X is calculated $\left[t_{X}^{m}-2\sigma ;\;t_{X}^{m}+2\sigma \right]$. This temporary-blocking window is calculated for the probabilistic distribution of the aircraft n based on its 4DT temporary windows.
4. Once the whole temporary-blocking window at the waypoint X is calculated for the aircraft m, this value is extrapolated to the departing time: $\left[t_{dep_{ini}}^{m};t_{dep_{fin}}^{m}\right]$.

The temporary-blocking windows for departures do not mean that it is not possible for aircraft m to depart, but it means that there exists conflict probability associated with aircraft n. This process allows determining the conflict probability between both aircraft for specific departing times considering 4DT time windows. The conflict probability is calculated by the number of infringements detected based on the total number of simulations:

(11) $$P=\frac{No\;conflicts}{No\;simulations}$$

## 5. Results and Discussion

### 5.1. Description of Case Study

Strategic conflict management is applied to a case study between two city-pairs in Spain. The city-pairs are Sevilla (LESV)–Barcelona (LEBL) and Vigo (LEVX)–Murcia (LELC), which are separated by similar distance and aircraft operationally and statistically fly at similar flight levels. Table 1 provides the main parameters of the case study. The value of the different times is provided as minutes: seconds.

**Figure 4:**
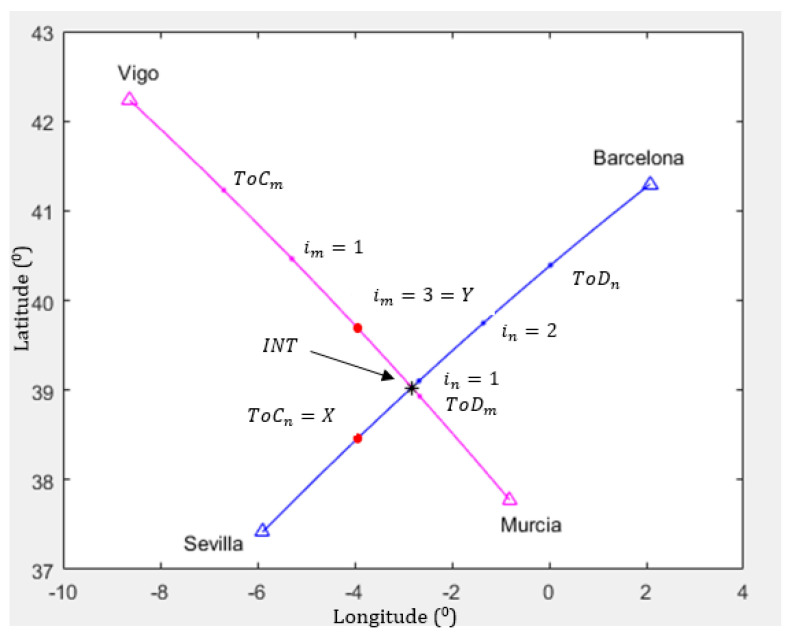
2D representation of case-study.

Figure 4 shows the 2D representation of the city-pairs Sevilla-Barcelona and Vigo-Murcia.

It can be observed at both trajectories that they are constituted by four waypoints at which the 4DT temporary restrictions must be fulfilled. The distance between the waypoints is l=100 NM. Waypoints X and Y (the last waypoints before the intersection) are highlighted in Figure 4, as well as the intersecting point. The geographic coordinates of these three points are:

$$X\{\begin{array}{c} \lambda_{X}=-3.9461\\ \varphi_{X}=38.4566 \end{array}\;\;Y\{\begin{array}{c} \lambda_{Y}=-3.9471\\ \varphi_{Y}=39.6966 \end{array}\;\;Int\{\begin{array}{c} \lambda_{int}=-2.822228\\ \varphi_{int}=39.028519 \end{array}$$

### 5.2. Conflict-Detection Management without Uncertainty

For the case study of Section 5.1, the temporary-blocking windows were calculated to the departures of the city-pair Sevilla–Barcelona. In this case, no 4DT uncertainty was considered, either wind or speed. The static temporary-blocking window for departures was [21:43; 24:34], a time span of 171 s. This was the baseline to be compared with the introduction of the probabilistic assessment.

### 5.3. Probabilistic Assessment

The next step was to introduce the uncertainty models described in Section 3 and to analyse how they modified the static temporary-blocking window. $10^{4}$ combinations assessed the integration of both trajectories because of the introduction of 4DT time windows.

#### 5.3.1. 4DT Uncertainty

**Figure 5:**
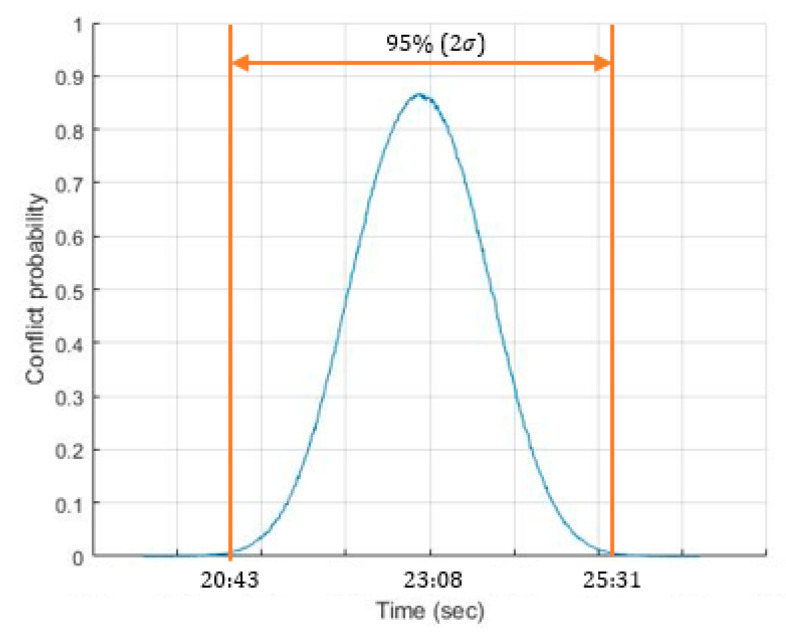
Conflict probability as a function of $t_{dep}^{Sevilla-Barcelona}$.

In this section, the 4DT time windows were applied to calculate the new temporary-blocking window. By applying the process described in Section 4, the new temporary-blocking window based on 4DT uncertainty was [20:43; 25:31] for the 95% (2σ), an increase of 112 s with the static temporary-blocking window. Figure 5 represents the probabilistic distribution obtained for the MC simulations. 

The conflict probability between both aircraft depended on the combination of both departing times. The average value of the conflict probability distribution is very high, exceeding 80% conflict probability. Therefore, it can be concluded that the introduction of 4DT trajectories (fulfilling temporary restrictions of 2 min at specific waypoints) can be introduced with a temporary-blocking window of 283 s to avoid conflict between this city-pair. This technique allows calculating the conflict probability based on 4DT and temporary requirements and the calculation of temporary-blocking windows for de-conflicting 4DT based on their departing times. The main concern of this study will be to ensure the application on the real operation and the validation of these results.

#### 5.3.2. Speed Uncertainty

As was explained in Section 3.2, two different modes of speed uncertainty were assumed to analyse their impact on 4DT: adjusted and updated speed. Table 2 provides the temporary-blocking windows associated with each mode.

The following conclusions could be extracted:

- The time span of the temporary-blocking window for the updated speed was 302 s, very similar to the 4DT uncertainty without speed uncertainty. Therefore, this technique increased the size of the temporary-blocking window and deteriorated the previous value of conflict-probability.
- The time span of the adjusted speed decreased to 166 s. This value was similar to the static case where no uncertainty was considered. The range of the speeds needed to correct the time windows were minor than 5%. This mode provided the most accurate results for conflict probability considering uncertainty. In this case, the pilot would adjust the speed once the aircraft reached a waypoint to ensure the time requirement of the following waypoint. The major limitation of this mode is that the aircraft had to modify its speed throughout the trajectory.

Therefore, the introduction of the adjusted-speed mode with 4DT improves the path predictability to the static temporary-blocking window. In other words, the requirement of adjusting the speed once a waypoint is reached provided the assurance of the same temporal requirements of the case without uncertainty. It could be possible the strategic conflict management depending on the temporary-blocking windows for departures by ensuring a requirement of adjusting the speed at the waypoints. However, the validation of these theoretical results for the path adherence could not be confirmed until 4DT will fly with these requirements.

#### 5.3.3. Wind Uncertainty

The last analysis focused on the way the wind uncertainty affects 4DT in terms of strategic conflict management. As was mentioned before, two different evaluations were performed depending on the wind that was considered as uniform throughout the scenario, or it varied in different geographical areas. 

##### Constant Wind

The first analysis assumed a constant wind throughout the trajectories. This simplification can be used in typical non-convective days where the wind variation is reduced. It was simulated a uniform wind for every direction, and the intensity varied from 0 to 40 knots (kts). These values were typical wind values that could appear for similar altitude conditions. Besides, wind direction varied every 20°. Table 3 shows the temporary-blocking windows for different cases.

From previous results, the temporary-blocking windows varied their time span and location for each combination of intensity and direction:

- The variation of the time span increased with the wind intensity, although the increase was limited until 3.1% (9 s). Therefore, the variation of the wind intensity below 40 kts can be neglected with respect to the size of the temporary-blocking windows with 4DT.
- However, the temporal displacement of the temporary-blocking windows cannot be neglected. Those variations could generate a delay or advance for the same wind direction up to 168 s of delay (wind direction 140°) and 138 s of advancement (direction 320°). The size of the 4DT temporary-blocking windows without speed uncertainty is 283 s (Section 5.3.1), this implied that there were temporal displacements over 40%. In the case the static temporary-blocking windows were considered, the displacement could reach up to 100%.

**Figure 6:**
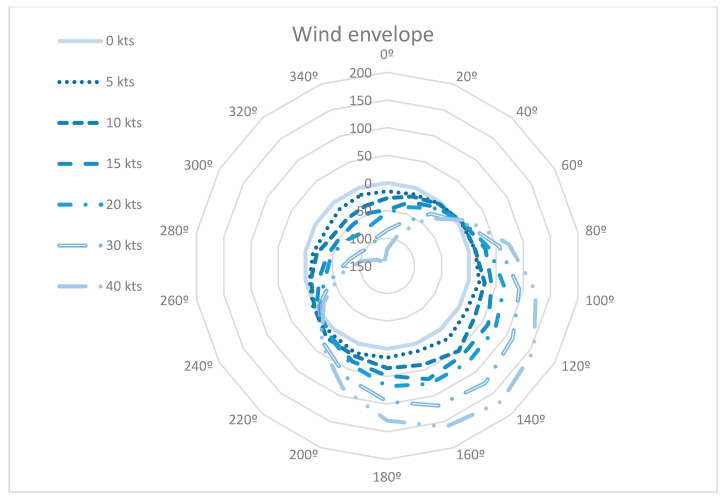
Envelope of temporary-blocking windows with 4DT and constant wind uncertainty.

Lastly, Figure 6 shows the variations of the temporary-blocking windows for the different combinations of intensity and direction compared with the reference case (0 kts). The temporary-blocking windows were delayed when the points are located outside of the envelope of 0 kts and advanced when they were inside of it. It was observed that the cross from advancement to delay was roughly obtained for 50° and 240°. Therefore, winds with directions from 50° to 240° advanced the temporary-blocking window and winds with directions from 240° to 50° delayed the temporary-blocking window. Nonetheless, these wind directions mainly depended on the city-pair selected and will affect differently for each city-pair.

According to the above results, the wind uncertainty affected the location of the temporary-blocking window but not their time span. To minimise the wind impact to the temporary-blocking windows, the wind direction should be known in advance with an accuracy of +/-20°. Therefore, the wind direction is a crucial factor for the calculation of temporary-blocking windows in strategic conflict management. 

##### Variable Wind

**Figure 7:**
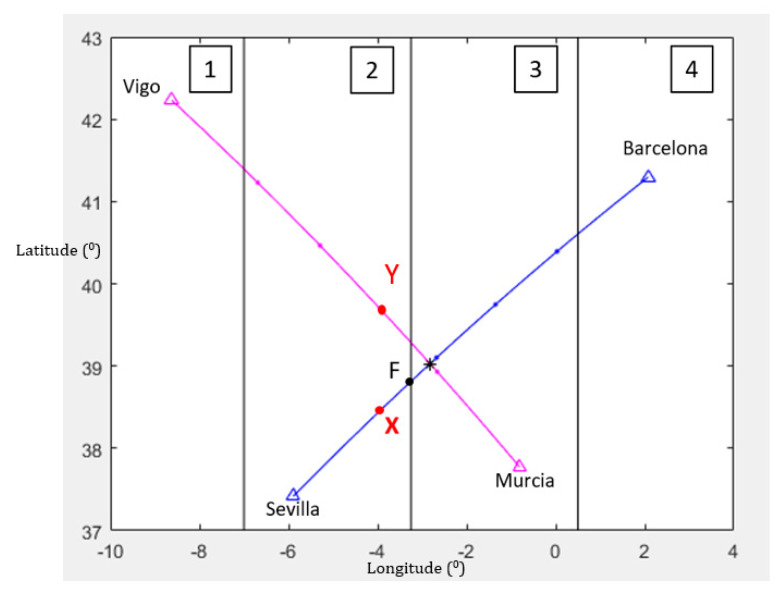
Limits of the areas where the wind varies.

The analysis for the influence of variable wind throughout the trajectories depended on the geographical areas they have flown. The airspace was divided into four geographical areas (see Figure 7) with a similar length around 100 NM where the wind typically does not exceed 10% variations. Hence, geographical areas 1 and 4 did not take part in the calculation of the uncertainty because the path stretch to study were located at geographical areas 2 and 3. Throughout geographical areas 1 and 4, the aircraft should take the necessary actions to ensure the 4DT time windows. 

The variation of the wind in geographical areas 2 and 3 was modelled with a variation of the direction of ±5° and intensity of ±10% from the geographical area 1. Although the wind variations were the same in module, the uncertainty was calculated independently for geographical area 2 and 3. 

Table 4 shows the results of the temporary-blocking windows for variable wind considering different wind directions. For the sake of clarity, Table 4 only shows the results for a nominal wind of 30 kts (which represents the worst scenario). The column difference indicates the variation in module of the case of variable wind compared with constant wind, and the column displacement indicates the variation of the location between the cases of variable wind compared with constant wind. 

The variation of the difference in module between the time span of the temporary-blocking windows did not exceed three seconds. Then, the influence of 10% wind variation between consecutive areas could be neglected. This result agreed with the results of the constant wind effect. About the displacement of the temporary-blocking windows, the effect of the variable wind implied a movement to both sides depending on the uniform wind. Moreover, this displacement did not exceed 5 s, the displacement could be neglected because it modified the size of the temporary-blocking windows less than 2%. However, it should be studied in further work the inclusion of a constant factor in order to take the wind variation into consideration.

Finally, these results concluded that the introduction of temporary-blocking windows could be a reliable and trustworthy system for strategic conflict management based on 4DT. The temporary-blocking windows were a powerful tool for air traffic management to ascertain departing restrictions between city-pairs. The implications could imply a reduction in the ATC workload by the reduction of conflict probability, but, it will demand to pilot modifications to ensure 4DT time windows that will mean pilot workload increase. Then, the model developed herein and the results achieved require further study. There are levels of error in the calculation of the temporary-blocking windows that should be reduced. On the one hand, the temporary-blocking windows were conservative because there were situations assumed as conflicts, but a more detailed assessment could indicate that there was no conflict. The strategic conflict management required knowledge of wind that cannot be ascertained at this strategic horizon; then, it is necessary to introduce new techniques to include more real wind conditions. Lastly further work will include the integration of temporary-blocking windows in the whole European path network; in other words, to tackle the multi-aircraft conflict-detection problem based on 4DT and the optimisation of the results. 

## 6. Conclusions

In this paper, the authors presented the results of a new methodology to deal with the strategic conflict management based on 4D trajectories, while taking a free-route scenario into account. The proposed methodology consisted of the calculation of conflict probability and temporary-blocking windows between two city-pairs. The temporary-blocking windows provided temporary time spans that blocked the departure of one aircraft because of the potential separation infringement in the air. The introduction of 4DT implied that aircraft must fulfil temporary restrictions at specific waypoints throughout the trajectories (4DT time windows). Moreover, the probabilistic assessment took into account the main uncertainties because of speed and wind effect. Speed was modelled based on two models: (1) Updated speed that recalculated the nominal speed based on the time the aircraft passes through the waypoint, and (2) adjusted speed that recalculated the nominal speed to fulfil the estimated average time at the next waypoint. The wind was modelled based on two assumptions as well: (1) The wind was constant throughout the trajectories, and (2) the wind varied throughout the trajectories depending on different geographical areas. The results of the probabilistic assessment were founded in $10^{9}$ Monte Carlo simulations for each experiment. Particularly, this research only considered conflict probability because it is the most widespread safety metric. The main concern of this safety metric is that it does not take into account the specific features of the separation infringements as the severity, duration or minimum distance. Although there is no unique and accepted safety metric, different safety metrics should be considered in further work to deepen in the characteristics of the conflicts, and to bring to the light new insights and methods to tune up conflict management. 

First, the length (time span) of the temporary-blocking windows increased with the introduction of uncertainty. The introduction of 4DT time windows implied a temporary-blocking window of almost 300 s while the static approach was 180 s. The introduction of the speed uncertainty provided different results depending on the mode analysed. Updated speed provided an increase of the temporary-blocking windows because of the uncertainty and modifications of the speed. However, adjusted mode reduced the temporary-blocking windows because the aircraft adjusted the speed to fulfil the estimated average time at the next waypoint. This case improved the results and reduced the uncertainty until similar values to the static case. Second, the introduction of wind did not influence the length of the temporary-blocking windows, although the variation of the intensity encompassed from 0 to 40 kts. However, the wind displaced the temporary-blocking windows by the advancement or delay from its original time location. This displacement was bigger depending on the wind direction and the wind intensity.

Therefore, the solution proposed in this work was the implementation of temporary-blocking windows between aircraft of different city-pairs. This solution would imply scheduling restrictions during the strategic phase based on 4DT, and it would demand a collaboration decision-making procedure between the airspace users, the ANSPs and the network manager. About the length of the temporary-blocking windows, the best solution demanded to minimise the conflict probability and the strategic conflict-management by the usage of the speed adjusted mode. This concept ensures that the pilot fulfils the temporary requirements for the 4D time windows, which, in turn, will increase the pilot workload because of the path modifications demanded. The main concern will be to ensure the application of this concept on the future real operation and the validation of these results. Further research should focus on the analysis of this study in the whole European network and the specifications to comply between AUs, ATC, ANSPs and NM. 

## Figures and Tables

**Table 1:** Parameters of the case-study.

City-Pair	Sevilla-Barcelona	Vigo-Murcia
**Departure**	5°53′35″ W, 37°25′05″ N	8°37′36″ W, 42°13′54″ N
**Arrival**	2°04′42″ E, 41°17′49″ N	0°48′45″ W, 37°46′30″ N
**Mach**	$v^{1}$ = 0.78	$v^{2}$ = 0.78
**Flight Level**	350	350
**Climbing time**	$t_{ToC}^{1}$ = 25:00	$t_{ToC}^{2}$ = 26:00
**Departure time**	-	$t_{dep}^{2}$ = 00:00
**Time at intersection**	-	$t_{int}^{2}$ = 47:00

**Table 2:** Temporary-blocking windows with speed uncertainty.

Mode	Temporary-Blocking Windows	Time Span (sec)
**Updated speed**	[20:36; 25:38]	302
**Adjusted speed**	[21:44; 24:30]	166

**Table 3:** Temporary-blocking windows for constant wind.

Wind Direction	Intensity (kts)						
	0	5	10	15	20	30	40
**0°**	[20:43; 25:31]	[20:29; 25:15]	[20:16; 25:03]	[19:54; 24:43]	[19:50; 24:38]	[19:18; 24:06]	[18:42; 23:32]
**20°**		[20:31; 25:19]	[20:26; 25:15]	[20:15; 25:05]	[20:05; 24:55]	[19:36; 24:26]	[19:13; 24:05]
**40°**		[20:40; 25:28]	[20:38; 25:28]	[20:31; 25:20]	[20:27; 25:18]	[20:14; 25:06]	[20:03; 24:55]
**60°**		[20:48; 25:37]	[20:49; 25:39]	[20:49; 25:41]	[20:50; 25:42]	[20:48; 25:42]	[20:55; 25:51]
**80°**		[20:54; 25:44]	[20:53; 25:45]	[21:12; 25:36]	[21:18; 26:10]	[21:35; 26:30]	[21:52; 26:49]
**100°**		[21:02; 25:50]	[21:11; 26:01]	[21:23; 26:14]	[21:45; 26:39]	[22:12; 27:07]	[22:42; 27:38]
**120°**		[20:57; 25:47]	[21:15; 26:06]	[21:43; 26:33]	[21:57; 26:49]	[22:32; 27:26]	[23:15; 28:12]
**140°**		[21:05; 25:53]	[21:32; 26:24]	[21:44; 26:35]	[21:59; 26:51]	[22:47; 27:40]	[23:31; 28:24]
**160°**		[20:56; 25:46]	[21:23; 26:12]	[21:49; 26:41]	[21:59; 26:49]	[22:41; 27:31]	[23:21; 28:13]
**180°**		[20:59; 25:47]	[21:18; 26:06]	[21:32; 26:20]	[21:51; 26:39]	[22:18; 27:07]	[22:53; 27:41]
**200°**		[21:00; 25:50]	[21:07; 25:54]	[21:17; 26:05]	[21:23; 26:11]	[21:49; 26:35]	[22:09; 26:55]
**220°**		[20:52; 25:40]	[21:03; 25:15]	[20:57; 25:43]	[20:58; 25:44]	[21:15; 26:00]	[21:20; 26:03]
**240°**		[20:47; 25:35]	[20:46; 25:32]	[20:41; 25:27]	[20:37; 25:21]	[20:38; 25:21]	[20:29; 25:10]
**260°**		[20:36; 25:23]	[20:34; 25:20]	[20:19; 25:04]	[20:09; 24:52]	[19:52; 24:36]	[19:42; 24:23]
**280°**		[20:29; 25:17]	[20:23; 25:09]	[20:04; 24:50]	[19:58; 24:42]	[19:24; 24:08]	[19:05; 23:46]
**300°**		[20:20; 25:10]	[20:06; 24:52]	[19:54; 24:39]	[19:43; 24:29]	[19:09; 24:51]	[18:38; 23:20]
**320°**		[20:27; 25:15]	[20:06; 24:52]	[19:51; 24:37]	[19:30; 24:16]	[18:56; 24:43]	[18:25; 23:09]
**340°**		[20:30; 25:18]	[20:09; 24:56]	[19:55; 24:42]	[19:34; 24:20]	[19:08; 24:56]	[18:26; 23:12]

**Table 4:** Temporary-blocking windows for constant and variable wind.

	Variable Wind	Difference		Displacement
**0°**	[19:23; 24:02]	2 sec	(0.7%)	−5 sec
**20°**	[19:54; 24:28]	0 sec	(0%)	2 sec
**40°**	[20:22; 24:04]	1 sec	(0.3%)	−2.5 sec
**60°**	[20:25; 24:46]	0 sec	(0%)	4 sec
**80°**	[21:26; 24:29]	0 sec	(0%)	−1 sec
**100°**	[22:32; 24:04]	−1 sec	(−0.3%)	−2.5 sec
**120°**	[22:28; 24:28]	−2 sec	(−0.7%)	3 sec
**140°**	[22:27; 24:38]	−1 sec	(−0.3%)	−1.5 sec
**160°**	[22:27; 24:31]	0 sec	(0%)	0 sec
**180°**	[22:27; 24:07]	−1 sec	(−0.3%)	0.5 sec
**200°**	[21:26; 24:35]	0 sec	(0%)	0 sec
**220°**	[21:25; 24:55]	−1 sec	(−0.4%)	−4.5 sec
**240°**	[20:25; 24:16]	1 sec	(0.4%)	−5.5 sec
**260°**	[19:54; 24:39]	−3 sec	(–1.1%)	4.5 sec
**280°**	[19:44; 24:11]	−2 sec	(−0.7%)	4 sec
**300°**	[19:23; 24:51]	1 sec	(0.4%)	−0.5 sec
**320°**	[18:53; 24:42]	0 sec	(0%)	−1 sec
**340°**	[18:23; 24:45]	−2 sec	(−0.7%)	−5 sec
